# Preliminary Investigation of Bovine Whole Blood Xenotransfusion as a Therapeutic Modality for the Treatment of Anemia in Goats

**DOI:** 10.3389/fvets.2021.637988

**Published:** 2021-03-04

**Authors:** Joe S. Smith, Austin K. Viall, Ryan M. Breuer, Rebecca A. Walton, Paul J. Plummer, Ronald W. Griffith, Amanda J. Kreuder

**Affiliations:** ^1^Veterinary Diagnostic and Production Animal Medicine, College of Veterinary Medicine, Iowa State University, Ames, IA, United States; ^2^Biomedical Sciences, College of Veterinary Medicine, Iowa State University, Ames, IA, United States; ^3^Veterinary Pathology, College of Veterinary Medicine, Iowa State University, Ames, IA, United States; ^4^Food Animal and Camelid Hospital, College of Veterinary Medicine, Iowa State University, Ames, IA, United States; ^5^Veterinary Clinical Sciences, College of Veterinary Medicine, Iowa State University, Ames, IA, United States; ^6^Veterinary Microbiology and Preventive Medicine, College of Veterinary Medicine, Iowa State University, Ames, IA, United States; ^7^National Institute of Antimicrobial Research and Education, Ames, IA, United States

**Keywords:** goat (*Capra aegagrus hircus*), anemia, transfusion, blood transfusion, xenotransfusion

## Abstract

Anemia requiring whole blood transfusion for appropriate treatment is a common clinical presentation of caprine patients to veterinary practitioners; however, identifying suitable blood donors in goat herds can be challenging. In other veterinary species, the practice of xenotransfusion, where blood from 1 species is transfused to another, is used in emergency settings. Due to their ability to donate large volumes of whole blood, cattle could be an ideal source for xenotransfusion of goats. In this study 2 healthy goats were transfused with bovine whole blood. The goats were then monitored for adverse effects and the presence of bovine erythrocyte post-xenotransfusion. Afterward, 15 caprine–bovine combinations were evaluated for compatibility via cross-matching. Both goats tolerated xenotransfusion, although transient reactions were observed. Of the 15 cross-match combinations, 11 of the major cross matches were compatible, and all minor cross matches were also compatible. While future work is necessary to refine this technique, xenotransfusion of goats with cattle blood may be a therapeutic modality for the treatment of caprine anemia.

## Introduction

Anemia is a common and challenging clinical condition to treat in goats. While anemia is a common sequelae of gastrointestinal parasite infections (1), commercially available caprine blood products are not currently available in the United States. Some tertiary care large-animal veterinary clinics have access to caprine whole blood through on-site donor animals; however, not all clinics can maintain this labor-intensive resource. Therefore, when anemic animals are identified by practitioners, the use of other animals from the same herd on an as-needed bases as blood donors is often necessary (2). However, in a herd situation, finding a donor can be extremely challenging, as anemia from gastrointestinal parasites is often a herd issue, making finding a donor with appropriate red blood cell concentrations difficult to find in herds with widespread parasitism. Unlike companion animal medicine, where access to non-pregnant and non-lactating animals is readily available, in production animal settings, pregnant and/or lactating animals constitute the majority of the herd. The use of a pregnant donor could be less than ideal for multiple reasons (3), which further impair donor identification in production settings. Additionally, the volume of blood available for donation is based on donor body weight, which may complicate matters further when only small amounts can be harvested from potential donors of smaller body size than the recipient. Due to these limitations, therapeutic options for anemia in goats are problematic because finding a suitable donor can be challenging, and obtaining the necessary volume may not be achievable from a single donor once identified.

Xenotransfusion is defined as the administration of blood or blood products from 1 species to another, and the roots of this practice were first reported in 1667 AD (4). This practice is used successfully in small animal emergency situations, as canine blood can be successfully transfused into feline patients for emergency use (4–7). Typically limited to one-time administration, xenotransfusion is a useful technique when a donor of the same species cannot be found (8). Reports in the literature are lacking on the administration of blood from cattle origin to goats. One study conducted by Mammerick et al. evaluated the response of sheep and goats to administration of blood from cattle infected with bovine leukosis virus (9), with no adverse reactions observed from the administration of blood. However, that study lacked the blood volume and monitoring necessary to justify the use of this technique in clinically anemic patients. In an emergent situation requiring xenotransfusion, cattle may prove to be an ideal blood donor for goats. The ability of cattle to donate 10–15% of their blood volume (2), which when considering the average size of cattle compared to goats (i.e., 5–7 L of blood for a 600-kg bovine vs. 500–700 ml for a 60-kg caprine), would insure that more than the amount necessary for an anemic goat could be collected. In addition to the volume collected, bovine red blood cells are robust and demonstrate low complement-mediated lysis (10). Thus, the aim for our study was to determine the feasibility and safety of xenotransfusion of bovine blood for treatment of anemia in goats.

## Materials and Methods

This project was approved by the Institutional Animal Care and Use Committee of Iowa State University (protocols: 17-8558, and 19-229). The study was divided into 2 phases. The initial phase was a live animal “proof of concept” study. The second phase of the study was a laboratory phase focusing on cross-match compatibility of bovine and caprine whole blood.

### Live Animal Proof of Concept

Two live Boer does (aged 11 and 10 months; weighing 41.2 and 39.9 kg) were examined for health prior to xenotransfusion. The does were healthy, and had pre-xenotransfusion hematocrit and plasma protein of 21.4%/6.6 g/dl and 27.8%/7.0 g/dl, respectively. The does were acclimated to the hospital setting in the Food Animal and Camelid Hospital (FACH) of Iowa State University for 3 days prior to initiation of the study. During the study, the goats were fed a diet of a commercial goat pellet as well as alfalfa hay, which either met or exceeded the National Research Council requirements for maintenance of goats at their status. Hay and water were provided *ad libitum* throughout the study.

One hour prior to xenotransfusion, 1 L of whole blood was collected from a multiparous 9-year-old Angus cow in normal health, with no recent history of drug administration. The cow was not currently pregnant or lactating, and had a packed cell volume/total solids of 31%/6.7 g/dl. An intravenous catheter was placed for collection of whole blood into a single 1-L commercial blood collection bag (Dry blood collection bag, J-520D, Jorgensen Laboratories, Loveland, Colorado). The collection bag was pre-treated with 143 ml of anticoagulant (anticoagulant citrate dextrose-A, Terumo BCT, Lakewood, CO). Afterward, the cow was administered a 1-L bolus of normal saline to replace the blood volume lost. During this time, the goats were clipped, prepped, and catheterized with a 3.25-inch, 14-gauge catheter (Milacath-Extended Use, MILA International Inc., Florence, Kentucky) in the jugular vein as previously described (11). Pre-transfusion temperature, heart rate, and respiratory rates were collected. The goats were pre-medicated with a 1.1-mg/kg dose of flunixin meglumine administered intravenously.

The transfusion was started at a rate of 1 ml/kg/h for the first 15 min, then, the rate was doubled to 2 ml/kg/h for the next 15 min. At 30 min, the rate was doubled again to 4 ml/kg/h, and then was doubled one last time at 60 min to a rate of approximately 8–10 ml/kg/h. Temperature, pulse, and respiratory rates were collected at 5, 10, 15, 30, 45, 60, 75, 90, and 105 min post initiation of the transfusion. During the xenotransfusion procedure, the does were closely monitored for signs of transfusion reactions.

Complete blood counts were collected pre-transfusion, as well as 1 and 4 days post-transfusion. Blood films were evaluated by a board-certified veterinary pathologist for relative count of bovine erythrocytes per 500 erythrocytes. As the mature bovine erythrocyte is approximately 5–6 μm in diameter (12) and the mature caprine erythrocyte is one of the smallest in domestic herbivores at approximately 3.39 μm in diameter (13), erythrocyte speciation is evident microscopically based on diameter as bovine erythrocytes are approximately 50% larger than caprine erythrocytes. Serum biochemistry results (BUN: Blood Urea Nitrogen; Creatinine; Total Bilirubin) were evaluated pre-transfusion, as well as 1 and 4 days post-transfusion by a validated biochemistry analyzer (14) at the Iowa State University Veterinary Clinical Pathology Laboratory. After the transfusion the goats were monitored for a 12-day period. At the end of the 12-day observational period, they were humanely euthanized and submitted for gross and histopathologic postmortem examination.

### Laboratory Phase

Under informed consent, blood samples were collected as convenience (*n* = 15 of each species) samples from inpatient cattle and goat patients, unrelated to the first phase, at the FACH. These samples were submitted within 2 h of each other and sent to the Iowa State University Clinical Pathology Laboratory for cross-matching of each caprine-bovine combination. One ml of EDTA whole blood from the donor bovine and recipient goat was centrifuged at 1,000 × *g* for 1 min, with plasma and erythrocytes subsequently separated. The erythrocytes were washed by resuspending in 2 ml of blood banking saline (Blood Bank Saline 0.85% NaCl, EKI Chemical. St Joliet, IL), centrifugation at 1,000 × *g* for 1 min, and then removal of saline; this process was repeated 4 times. A 5% working stock suspension of erythrocytes was subsequently made by combining 1 drop of concentrated erythrocytes (RBCs) and 19 drops of blood banking saline. The following combinations were then made: donor control (1 drop of donor RBCs with 2 drops of donor plasma), recipient control (one recipient RBCs with 2 drops of recipient plasma), major crossmatch (1 drop of donor RBCs with 2 drops of recipient plasma), and minor crossmatch (1 drop of recipient RBCs with 2 drops of donor plasma). These combinations were incubated at 37°C for 15 min. Samples were then centrifuged at 1,000 × *g* for 45 s and then analyzed for compatibility.

For compatibility evaluation of each combination, the plasma in the centrifuged tube was examined grossly for hemolysis. The erythrocyte pellet was then gently agitated to evaluate for macroscopic agglutination, and then the sample was examined microscopically following saline wash. The macroscopic agglutination was scored as follows: 4+ incompatible reaction—one or two solid clumps with clear plasma, 3+ incompatible reaction—few to several large clumps with clear plasma, 2+ incompatible reaction—many medium-sized clumps with clear to cloud background, 1+ incompatible reaction—numerous small clumps, barely visible macroscopic aggregates with cloudy background, incompatible ±, erythrocyte aggregates observed on microscopic evaluation only, and compatible, no macroscopic or microscopic agglutination detected. Autoagglutination observed in the donor or recipient controls nullified all test results.

## Results

### Live Animal Proof of Concept

For the goats utilized in phase one of this study, physical examination, serum biochemistry analysis, and complete blood counts were all within normal limits prior to the study with the exception of increased bilirubin in both does and mildly decreased hematocrit in 1 doe (Supplementary Table 1). The first doe received 300 ml of bovine whole blood. Mild evidence of transfusion reaction was observed for the first doe including hyperemia around the vulva, urticaria of the ears, piloerection, and loose stool at the conclusion of the transfusion, which lasted 105 min. The doe was bolused 400 ml of mixed polyionic intravenous fluids (Normosol-R, ICU Medical, Lake Forest, IL) for isotonic volume replacement, following the conclusion of the transfusion and the abnormal findings resolved shortly afterward (~20 min after initial observation).

The second doe received 319 ml of bovine whole blood. At 75 min into the transfusion, the doe was noted to be agitated and restless, repeatedly rising and lying down. Piloerection was noted, and swelling was observed along the muzzle and skin of the eyelids. Loose stool was passed, and an increased respiratory rate (72 breaths/min) was detected. At this 75 min mark, the transfusion was discontinued, and a 400 ml mixed polyionic fluid bolus, as well as 0.01 mg/kg of epinephrine were administered via IV route. The doe resumed normal behavior ~30 min after the administration of fluids and epinephrine and was observed eating 1 h after cessation of xenotransfusion. The next day, the muzzle swelling had resolved.

Normal clear urination and pelleted stool were observed from both does after the first day of observation following the xenotransfusion. Urinalysis was negative for blood during follow up. Serum biochemistry performed the day after xenotransfusion revealed an increased creatinine concentration for the first doe [1.0 mg/dl; reference interval: (0.3–0.8 mg/dl)] and a mild azotemia for the second doe [BUN: 77 (reference interval: 19–34), as well as creatinine: 2.2 mg/dl]. The values returned to within-normal reference intervals for the second doe within 48 h without further treatment and were not rechecked for the first. The transfusion results and serum biochemistry results are available in Supplementary Tables 1, 2, respectively.

**Figure 1:**
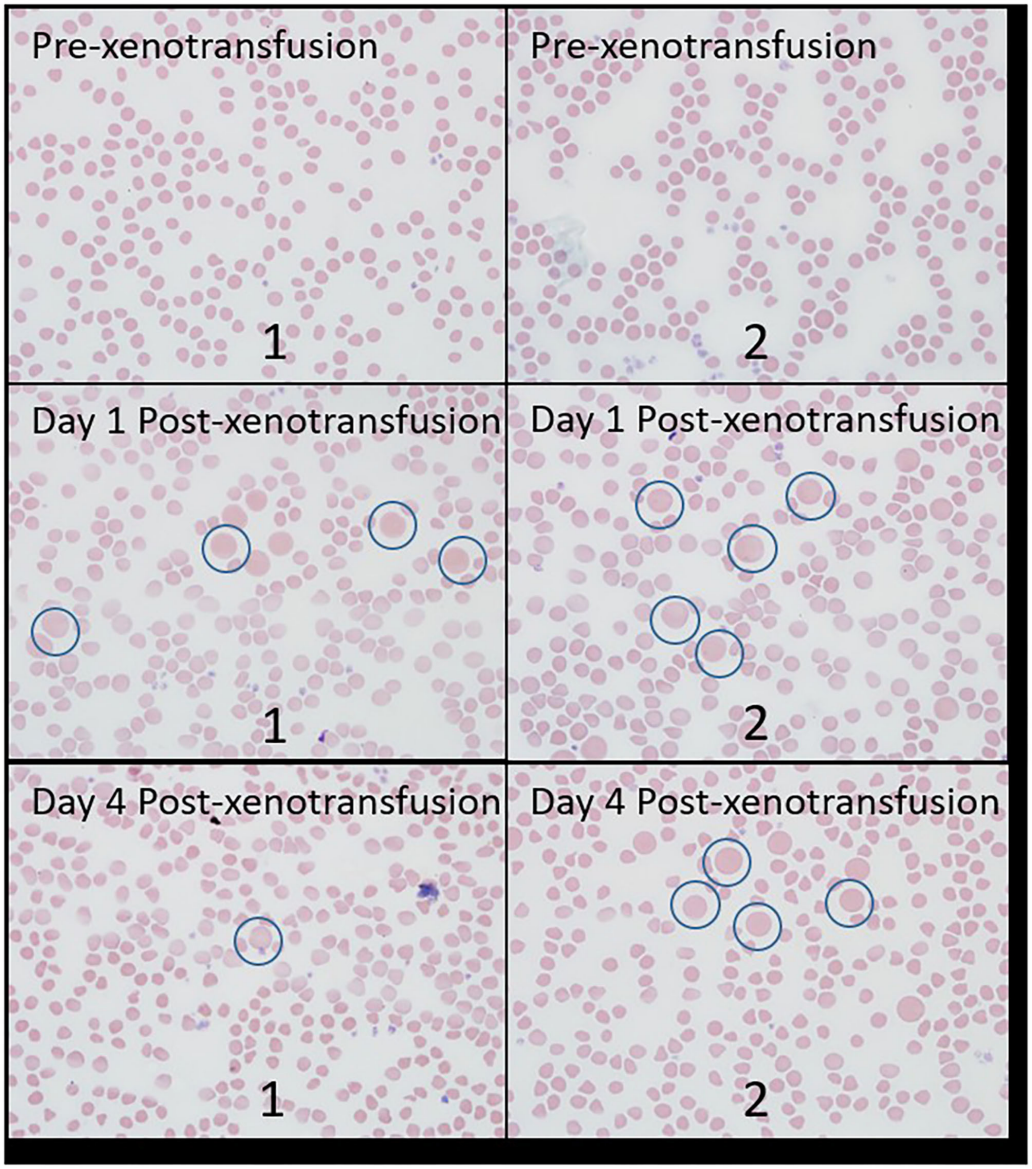
Blood films from both goats from samples collected pre-xenotransfusion, day 1 post-xenotransfusion, and day-4 post xenotransfusion. Examples of erythrocytes of size consistent with bovine erythrocytes are circled in blue. “1”: goat 1; “2”: goat 2.

The relative count of bovine erythrocytes per 500 erythrocytes is displayed in Table 1. Erythrocytes of sizes consistent with bovine erythrocytes were not noted prior to xenotransfusion in either doe, but were observed at days 1 and 4 post xenotransfusion in both does. Figure 1 displays an example of blood films from both goats, demonstrating caprine, and bovine erythrocytes. No abnormalities were noted on gross or histologic postmortem examinations for either doe.

**Table 1:** Number of erythrocytes of bovine morphology per 500 erythrocytes counted.

**Identification**	**Pre-xenotransfusion**	**Day 1 post-xenotransfusion**	**Day 4 post-xenotransfusion**
Goat 1	0	8	1
Goat 2	0	9	5

### Laboratory Phase

Fifteen cattle and goat cross matches were performed. Eleven of the 15 (73.3%) pairings had compatible major cross matches. All minor cross matches (donor serum matched with recipient red blood cells) were compatible. The results of these cross matches are listed in Table 2. All animals were utilized once with the exception of an Alpine wether and Angus bull, which each provided samples on 2 separate occasions.

**Table 2:** Macroscopic Agglutination Score: 4+ incompatible reaction–one or two solid clumps with clear plasma; 3+ incompatible reaction–few to several large clumps with clear plasma; 2+ incompatible reaction–many medium-sized clumps with clear to cloud background; 1+ incompatible reaction–numerous small clumps; barely visible macroscopic aggregates with cloudy background; incompatible ±, erythrocyte aggregates observed on microscopic evaluation only; and compatible, no macroscopic or microscopic agglutination detected (displayed as “ns,” as no agglutination was seen).

**ID**	**Recipient**	**Donor**	**Major cross match**	**Macroscopic agglutination score**	**Minor cross match**	**Auto-control**
1	Boer buck	Mixed breed beef steer	Incompatible	2+	Compatible	–/–
2	Alpine wether	Angus bull	Compatible	ns	Compatible	–/–
3	Boer doe	Mixed breed beef bull	Compatible	ns	Compatible	–/–
4	Boer doe	Mixed breed beef heifer	Compatible	ns	Compatible	–/–
5	Boer doe	Angus bull	Incompatible	1+	Compatible	–/–
6	Boer doe	Shorthorn heifer calf	Incompatible	2+	Compatible	–/–
7	Boer buck kid	Holstein bull calf	Compatible	ns	Compatible	–/–
8	Boer doe	Holstein bull calf	Incompatible	2+	Compatible	–/–
9	Toggenburg doe	Holstein bull calf	Compatible	ns	Compatible	–/–
10	Toggenburg doe	Holstein bull calf	Compatible	ns	Compatible	–/–
11	Toggenburg doe	Holstein bull calf	Compatible	ns	Compatible	–/–
12	Toggenburg doe	Holstein bull calf	Compatible	ns	Compatible	–/–
13	Toggenburg doe	Holstein bull calf	Compatible	ns	Compatible	–/–
14	Alpine wether	Angus bull	Compatible	ns	Compatible	–/–
15	Boer doe	Mixed breed beef bull	Compatible	ns	Compatible	–/–

*Pairing information and cross match results from the 15 caprine–bovine combinations used from this study. Note all animals were utilized once except for the Alpine wether in pairings 2 and 14, and the Angus bull in pairings 5 and 14*.

## Discussion

Xenotransfusion has been successfully described in multiple veterinary species for emergency situations (8, 15–17). Many species differ in blood types and presence of naturally occurring alloantibodies. These limitations have led to the exploration and use of xenotransfusion in certain species. Due to the lack of availability of feline type B blood and the presence of natural alloantibody production in cats, there are several documented cases of cats being transfused with canine blood, with the first study of the practice being reported by Hessler et al. (18), Euler et al. (6), and Le Gal et al. (16). More recently, a 13 month old Sphinx cat was transfused with 131 ml of canine blood due to hemorrhage from the caudal intercostal artery during patent ductus arteriosus ligation (19). Ultrasound-guided intracardiac xenotransfusion of canine packed red blood cells was used in emergency resuscitation of an 8-year-old cat with severe anemia from a massive flea infestation (7). Canine whole blood was also used for emergency resuscitation of an Island Fox after rattlesnake envenomation (17). These cases of canine xenotransfusions were successful. Xenotransfusion techniques are not limited to small animals, as recent xenotransfusion with bovine erythrocytes was used in a 4 month-old wildebeest calf (*Connochaetes taurinus*) undergoing a bilateral pelvic limb fracture repair (15). The animal was pre-medicated with prednisolone, and the technique allowed for stabilization from mass intraoperative hemorrhage that occurred during the fracture repair.

While xenotransfusions have a proven role in the emergent setting, adverse effects have been reported from xenotransfusion in other species. In the Hessler study, compatibility was assessed by slide agglutination and *in vitro* hemolysis testing prior to canine whole blood administration and after administration of 5–50 ml of canine whole blood. No adverse effects were noted initially; however, positive slide agglutination testing was noted 4–7 days afterward (18). When these cats were transfused again, all but one displayed signs of anaphylaxis, and fatalities were observed (18). The Sphinx cat did not display any evidence of hemolysis or anemia after the transfusion (19) and neither did the cat undergoing the ultrasound-guided procedure (7). In a recent retrospective study of 49 canine whole blood to feline transfusion patients, 10 died or were euthanized within 24 h, and a hemolytic transfusion occurred in 63% of the cats (16). That study also identified a febrile non-hemolytic reaction in 12.2% of the cats (16), which was similar to the 10% rate previously reported by another study (20). Azotemia post-xenotransfusion is another adverse effect that has been noted in small animal species (5). Among 22 cats that underwent xenotransfusion with canine blood, seven were azotemic after transfusion administration (16). Icterus and delayed hemolysis have also been reported in feline patients receiving canine blood (5, 16). Hyperbilirubinemia was observed in a domestic ferret transfused with type A feline packed red blood cells (21). Overall, there are several complications associated with xenotransfusions including hemolytic reactions, febrile transfusion reactions, and azotemia, and hyperbilirubinemia.

When considering xenotransfusion for goats, clinicians should consider the risk of adverse effects. One of the does in our study displayed azotemia, although it was transient in nature and resolved several days post transfusion. Icterus and hemolysis were not observed in the does in our study, but are possible. None of the goats in our study were febrile, as has been reported in cats following the xenotransfusion (16). However, the pretreatment with flunixin meglumine may have prevented hyperthermia for these does. While case reports describe premedication prior to transfusion in goats (22), and the practice is common in large animal medicine, evidence for premedication with a non-steroidal is lacking in small animal medicine. Delayed transfusion reactions could also occur, although they were not observed in our study. It should be noted, however, that blood transfusion within species is not without risk in small ruminants, as a recent study identified adverse reactions occurring 16% of the time in small ruminant blood transfusions (23). Bovine blood may also present an infectious disease risk, exemplified by bovine leucosis virus transmission (9). One small animal practice for relaying these risks to clients is verbal presentation followed by a written risk assessment (16). Strategies for reducing risk of adverse effects from xenotransfusion could include premedication as well as frequent monitoring post transfusion. Ambulatory practitioners could benefit from the development of a stall-side cross-matching test for xenotransfusion, similar to the modified rapid gel assay proposed for horses in the field (24). In small animal medicine, the antihistamine diphenhydramine is the drug commonly used to prevent acute febrile and allergic transfusion reactions (25). Currently, there are no pharmacokinetic studies describing diphenhydramine in goats, although there are references among ruminant species for sheep (26) and camels (27). Future work should consider evaluating the effect of an antihistamine on reactions from xenotransfusion in goats (28).

Interestingly, of the 2 pairings that utilized a sample from the Angus bull, both an incompatible and compatible cross match result was obtained. This could be due to a myriad of reasons depending on the matched goat's blood type and previous antigen exposure. Another potential cause of unknown probability could be the presence of a transient blood type, as has been reported in the cat that reverted from type AB to type B blood (29). It is currently unknown if transient blood types occur in ruminant species. While cross matching should be utilized prior to transfusions, clinicians should keep in mind that it may not be a complete surrogate for *in vivo* compatibility. In other large animal species cross match incompatibility is predictive of transfusion reactions and shortened red blood cell lifespan (30). With this in mind, an incompatible cross match should in theory eliminate a donor-recipient pair for xenotransfusion.

Limitations of our study include the preliminary nature and the small number of research subject used. For the live animal study, cross-matching for goats was not available at the author's institution (ISU) at the time, so it was not performed, essentially mimicking field conditions. It is possible that one or both of the does were incompatible with the bovine donor. In addition to the lack of cross-matching performed, the donor and the recipient does were not blood typed. Future research should consider the effect of blood type on outcome in large animal xenotransfusion. Cats have 3 main blood groups (31, 32), and over 13 blood groups have been described for dogs (33). Erroneous blood typing in 2 cats recently led to non-fatal acute hemolytic reactions following xenotransfusion with canine blood (6). Currently, there are thought to be 11 genetic systems of blood groups in cattle, and at least 6 in goats; however, there are no commercial ruminant blood typing services available in the United States (2). In addition to limitations associated with typing and cross-matching, size differences in bovine and caprine ruminant erythrocytes have been previously documented (13); however, it is possible, although unlikely, that the presumptive bovine erythrocytes noted at days 1 and 4 post-transfusion were caprine cells of considerably larger than normal size. However, no erythrocytes of this size were noted in either goat prior to the study. Future studies should evaluate erythrocyte kinetics via other methods, such as biotin-tagging and flow cytometry, as this method would allow for more precise evaluation of erythrocyte longevity (34). Future studies should also consider other ruminant species as blood donors for xenotransfusion. Sheep possess an erythrocyte that is more closely related to cattle in size than the goat erythrocyte, as well as more blood groups. Size disadvantages aside, goats may be ideal donors for some situations, as goat blood is less antigenic than either sheep or cattle blood due to the lack of R and J factors (10, 28).

The results of our preliminary study suggest that xenotransfusion with bovine whole blood can be tolerated by goats. Similarly, cross-matching results from our study indicate that some bovine to caprine blood transfusions would be compatible. Further investigation is necessary to refine the technique for this emergency practice, as practitioners/clinicians and clients should be aware of the potential adverse effects of xenotransfusion, as reported in other species, as well as within this study, when considering transfusing cattle blood to goats.

## Data Availability Statement

The original contributions presented in the study are included in the article/Supplementary Material, further inquiries can be directed to the corresponding author/s.

## Ethics Statement

The animal study was reviewed and approved by Institutional Animal Care and Use Committee, Iowa State University. Written informed consent was obtained from the owners for the participation of their animals in this study.

## Author Contributions

JS conceived the project. JS, AK, and RB performed the live animal portion of the study. JS, AK, PP, RG, and AV completed the *in vitro* portion of the study. RW provided conceptual clinical support to study design. All authors contributed to manuscript construction.

## Conflict of Interest

The authors declare that the research was conducted in the absence of any commercial or financial relationships that could be construed as a potential conflict of interest.
