# Supplementary material for: Preliminary Investigation of Bovine Whole Blood Xenotransfusion as a Therapeutic Modality for the Treatment of Anemia in Goats
Source: Front Vet Sci. 2021 Mar 4;8:637988. doi: 10.3389/fvets.2021.637988 (PMC7969644; doi:10.3389/fvets.2021.637988)
Supplement: Supplementary file 1 [file Table_1.DOCX]

**Supplemental Table 1**: Temperature, Pulse, and Respiration results pre-xenotrasnfusion (Time 0), and throughout the xenotransfusion procedure for 2 goats.

| Goat | Time (minutes) | Xenotransfusion  Rate (mL/kg/hr) | Temperature  (° C) | Pulse  (beats/minute) | Respiration (breaths/minute) |
| --- | --- | --- | --- | --- | --- |
| 1 | 0 | 0 | 39.2 | 80 | 48 |
| 1 | 5 | 5 | 39.3 | 80 | 42 |
| 1 | 10 | 5 | 39.2 | 90 | 48 |
| 1 | 15 | 5 | 39.3 | 80 | 40 |
| 1 | 30 | 10 | 39.3 | 90 | 48 |
| 1 | 45 | 20 | 39.1 | 80 | 36 |
| 1 | 60 | 20 | 39.3 | 90 | 42 |
| 1 | 75 | 20 | 38.9 | 120 | 40 |
| 1 | 90 | 20 | 39.0 | 80 | 48 |
| 1 | 105 | 20 | 38.9 | 60 | 36 |
| 2 | 0 | 0 | 39.4 | 80 | 40 |
| 2 | 5 | 5 | 39.5 | 90 | 40 |
| 2 | 10 | 5 | 39.5 | 80 | 40 |
| 2 | 15 | 5 | 39.6 | 84 | 40 |
| 2 | 30 | 10 | 39.4 | 80 | 40 |
| 2 | 45 | 20 | 39.2 | 90 | 40 |
| 2 | 60 | 20 | 39.1 | 100 | 36 |
| 2 | 75 | 29 | 39.2 | 80 | 72 |
