# Supplementary material for: Preliminary Investigation of Bovine Whole Blood Xenotransfusion as a Therapeutic Modality for the Treatment of Anemia in Goats
Source: Front Vet Sci. 2021 Mar 4;8:637988. doi: 10.3389/fvets.2021.637988 (PMC7969644; doi:10.3389/fvets.2021.637988)
Supplement: Supplementary file 2 [file Table_2.DOCX]

**Supplemental Table 2**: Serum Blood Urea Nitrogen (BUN); Creatinine, Total Bilirubin, Hematocrit and Plasma Protein concentrations of the study goats prior to xenotransfusion (day 0), 24 hours afterward (day 1) and 96 hours afterwards (day 4). Note: na (not assessed)

|  | Units | Day 0 | Day 1 | Day 4 | Normal Reference Range |
| --- | --- | --- | --- | --- | --- |
| Goat 1 |  |  |  |  |  |
| BUN | mg/dL | 22 | 29 | na | 19-34 |
| Creatinine | mg/dL | 0.8 | 1 | na | 0.3-0.8 |
| Bilirubin | mg/dL | 0.63 | 0.53 | na | 0.07-0.17 |
| Hematocrit | % | 21.4 | 22.8 | 23.5 | 24-48 |
| Plasma Protein | gm/dL | 6.6 | 6.9 | 7.3 | 6.0-7.5 |
| Goat 2 |  |  |  |  |  |
| BUN | mg/dL | 24 | 77 | 20 | 19-34 |
| Creatinine | mg/dL | 0.8 | 2.2 | 0.8 | 0.3-0.8 |
| Bilirubin | mg/dL | 1.05 | 0.62 | na | 0.07-0.17 |
| Hematocrit | % | 27.8 | 25.6 | 25.7 | 24-28 |
| Plasma Protein | gm/dL | 7.0 | 7.2 | 7.2 | 6.0-7.5 |
